# Granular parakeratosis associated with the use of laundry sanitizers containing benzalkonium chloride: a case report

**DOI:** 10.3389/falgy.2026.1906480

**Published:** 2026-08-26

**Authors:** Meizhen Fang, Jingqiu Zhang, Zilu Qu, Bin Hu, Yun Xia, Liang Zhang, Liuqing Chen

**Affiliations:** 1 Department of Dermatology, Wuhan No. 1 Hospital, Wuhan, China; 2 Hubei Province Key Laboratory of Skin Infection and Immunity, Hubei Provincial Clinical Research Center for Infectious Skin Diseases, Wuhan, Hubei, China

**Keywords:** benzalkonium chloride, granular parakeratosis, intertriginous areas, laundry sanitizer, skin barrier dysfunction

## Abstract

**Background:**

Granular parakeratosis (GP) is a rare benign inflammatory dermatosis that preferentially involves the axillae and other intertriginous, friction-prone skin sites and typically presents with pruritic erythema and scaling. Cases associated with benzalkonium chloride (BAC) exposure have rarely been systematically summarized, and further clinical evidence is needed. Herein, we report a case of generalized GP potentially associated with long-term exposure to a BAC-containing laundry sanitizer, with the aim of expanding the clinical spectrum of cutaneous adverse reactions associated with BAC.

**Case presentation:**

A 43-year-old Asian man presented with a persistent rash on the trunk and intertriginous areas for more than 1 year. He had a long-standing history of eczema with possible underlying skin-barrier dysfunction but no confirmed diagnosis of atopic dermatitis. He had regularly used a commercial laundry sanitizer containing BAC (0.5 g/L) for clothing disinfection since June 2020, at a frequency of 1–3 times per week. The first rash appeared in July 2021, approximately 13 months after the start of exposure, initially involving the groin and subsequently spreading symmetrically to the neck, axillae, antecubital fossae, lateral waist, back (with sparing of the spinal midline), and undergarment-covered areas. Fungal microscopy and periodic acid–Schiff (PAS) staining were negative. Histopathology showed epidermal hyperkeratosis with characteristic granular parakeratosis, intracorneal pustule formation, neutrophil aggregation, and superficial dermal perivascular inflammatory infiltrates. Direct immunofluorescence for IgG, IgA, IgM, and C3 was negative. In August 2023, the patient completely discontinued the BAC-containing laundry sanitizer, repeatedly rinsed all garments with clean water, and used topical emollients for skin-barrier repair. The lesions gradually subsided and achieved complete clinical remission in September 2023, approximately 1 month after the intervention. No recurrence was observed during 1 year of follow-up.

**Conclusions:**

Exposure to BAC-containing laundry sanitizer may be a potential trigger for generalized GP in susceptible individuals. A history of eczema and associated skin-barrier dysfunction may increase susceptibility to irritant exposure. Avoidance of suspected irritants together with skin-barrier repair may be effective in the management of BAC-associated GP. Further prospective studies and confirmatory testing are needed to clarify the causal relationship.

## Introduction

Granular parakeratosis (GP) is an uncommon benign inflammatory dermatosis characterized histologically by hyperkeratosis with retained keratohyalin granules in the stratum corneum. It mainly affects intertriginous skin, including the axillae, groin, and inframammary areas (1). Clinically, GP typically manifests as erythematous, scaly, pruritic papules or plaques and may occasionally show pustular features, with exacerbation in hot and humid environments. Its pathogenesis remains incompletely understood and is generally considered to represent a reactive epidermal process associated with occlusion, excessive sweating, maceration, or external irritants (2).

With the widespread use of disinfectant products, GP associated with exposure to benzalkonium chloride (BAC) has been increasingly recognized (5, 7). BAC is a quaternary ammonium compound widely used as a disinfectant and preservative in laundry products, personal-care products, and medical solutions. However, published evidence largely consists of case reports and small case series. Herein, we report a case of generalized GP with a detailed history of exposure to a BAC-containing laundry sanitizer, clinical course, ancillary investigations, and treatment outcome, and discuss the relevant differential diagnoses and potential role of BAC exposure.

## Case presentation

A 43-year-old Asian man presented to our dermatology outpatient department with a more than 1-year history of recurrent and progressive skin lesions. The eruption initially appeared in the groin and gradually extended to the trunk and major intertriginous areas over approximately 6 months. During disease progression, the patient reported intermittent burning pain, local swelling, and mild pruritus. The lesions worsened markedly in summer with heat, sweating, and increased skin moisture, but improved during the cooler and drier autumn and winter months. In hot weather, the lesions were characterized predominantly by bright erythema with numerous pinpoint pustules. During periods of improvement, the erythema faded to brown hyperpigmented patches with thin brown scales.

The patient had a history of eczema, with occasional mild xerosis and pruritus, but had never received a definite diagnosis of atopic dermatitis. He had no other chronic systemic diseases, no history of chemotherapy or long-term use of relevant medications, and no family history of hereditary or autoimmune skin disease. Before presentation, he had intermittently used a topical compound corticosteroid ointment, which temporarily relieved the pruritus and erythema; however, the lesions rapidly recurred after treatment was discontinued. He also reported an unpleasant odor from the affected skin during heavy sweating, which affected his daily life and quality of life. General physical examination revealed no significant abnormalities.

Detailed exposure timeline: The patient began using a commercial laundry sanitizer containing BAC (0.5 g/L) in June 2020, generally 1–3 times per week. The first skin eruption appeared in July 2021, approximately 13 months after the start of exposure, initially involving the groin. Despite persistent and progressive lesions, he continued to use the sanitizer intermittently. In August 2023, he completely discontinued the BAC-containing laundry sanitizer, thoroughly rinsed all garments with clean water multiple times before wearing them, and consistently applied topical emollients for skin-barrier repair. The lesions gradually subsided and achieved complete clinical remission in September 2023, approximately 1 month after the intervention. No recurrence was observed during the 1-year telephone follow-up through September 2024.

**Figure 1:**
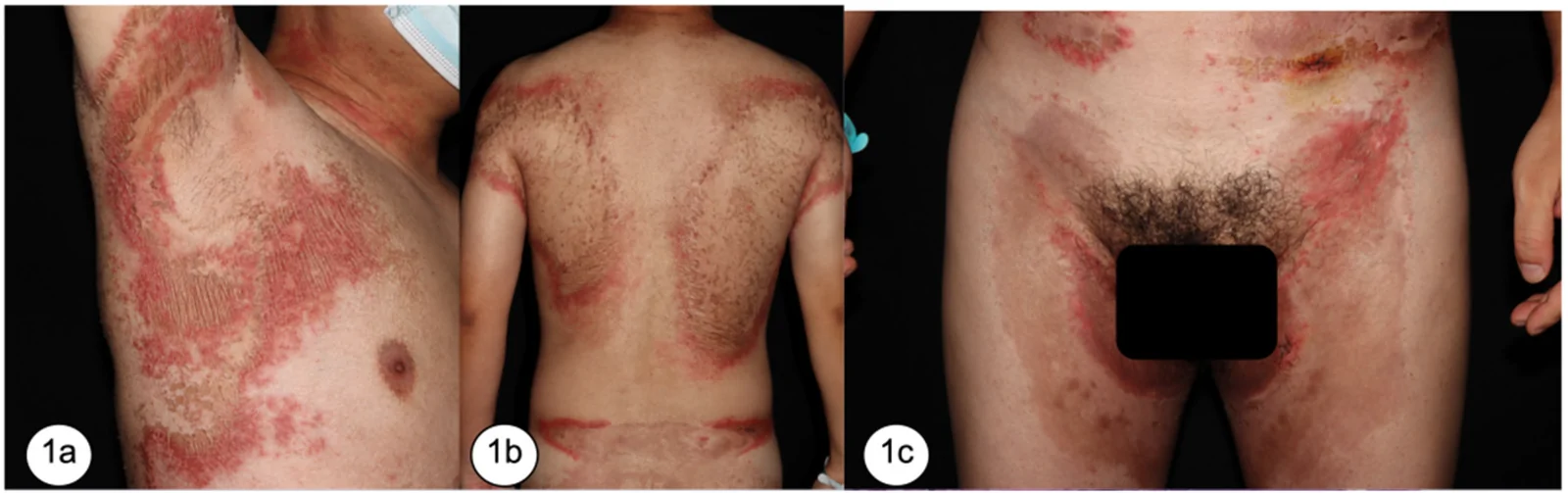
**a–c**. Clinical manifestations. **(a)** Axillary map-like erythema with scaling. **(b)** Symmetric erythema and pinpoint pustules on the back. **(c)** Hyperpigmented erythema with superficial scaling in an undergarment-covered area.

Dermatological examination showed symmetrically distributed lesions involving the neck, bilateral axillae, antecubital fossae, lateral waist, paravertebral back (with sparing of the spinal midline), and undergarment-covered areas. The lesions consisted of irregular map-like annular erythema with central hyperpigmentation, superficial leaf-like scaling, and numerous pinpoint pustules on the erythematous base (Figures 1a–c).

**Figure 2:**
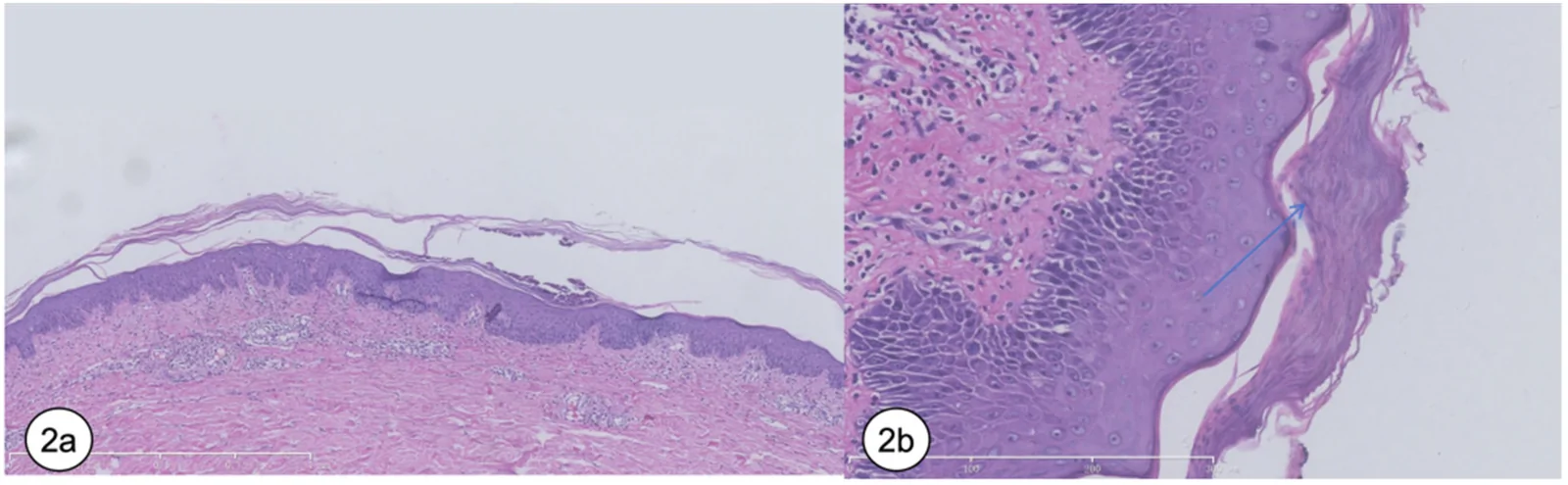
**a,b**. Histopathological features (hematoxylin and eosin staining). **(a)** Low-power view (100×) showing epidermal hyperkeratosis, granular parakeratosis, and superficial dermal perivascular inflammatory infiltration. **(b)** High magnification (200×): Intra-corneal pustule formation with massive neutrophil aggregation and retained keratohyalin granules in the stratum corneum. Arrows indicate typical granular parakeratosis changes.

Fungal microscopic examination was performed on scales scraped from active lesions, and no fungal hyphae or spores were identified. PAS staining of lesional tissue was also negative, providing no evidence of superficial fungal infection. A skin biopsy was obtained from an active erythematopustular lesion in the inguinal region. Hematoxylin and eosin staining showed marked epidermal hyperkeratosis, characteristic granular parakeratosis with retained keratohyalin granules in the stratum corneum, intracorneal pustule formation with prominent neutrophil aggregation, and superficial dermal perivascular inflammatory infiltrates composed predominantly of lymphocytes with scattered neutrophils (Figures 2a,b). Direct immunofluorescence showed no deposition of IgG, IgA, IgM, or C3 in the epidermis or dermis. Based on the clinical presentation, negative fungal studies, and characteristic histopathological findings, a diagnosis of granular parakeratosis was made.

Following diagnosis, the patient continued strict avoidance of BAC-containing laundry disinfectants, enhanced rinsing of clothing, and regular topical emollient therapy for skin-barrier repair. No systemic medication or topical corticosteroid was used. All lesions resolved completely within approximately 1 month, and no recurrence occurred during the 1-year follow-up.

## Discussion and conclusions

GP is a rare benign skin condition that most commonly affects the axillae and other intertriginous areas and is frequently associated with pruritus. Although its etiology remains uncertain, GP is generally regarded as a reactive epidermal process (1). Reported precipitating factors include occlusion (2), warm environments (3), sweating, obesity, frequent washing (4), and exposure to external irritants such as antiperspirants, deodorants, and zinc oxide. BAC, a preservative and antimicrobial agent present in numerous products including laundry products, personal-hygiene wipes, and medical solutions, has also been associated with GP (5–7).

Previous studies have suggested that atopic dermatitis may predispose patients to GP because epidermal-barrier dysfunction may facilitate penetration of irritants (8, 9). Although the present patient had a long-standing history of eczema rather than confirmed atopic dermatitis, impaired skin-barrier function may have increased susceptibility to repeated irritant exposure. Residual BAC on clothing may have provided repeated cutaneous exposure. As a cationic surfactant, BAC may disrupt epidermal barrier function and interfere with keratinocyte maturation (5, 6). Heat, sweating, moisture, and friction may further promote maceration and irritation, potentially accounting for the marked seasonal fluctuation observed in this patient.

Physical factors such as hyperhidrosis, obesity, and friction have also been proposed to contribute to GP by impairing epidermal maturation and barrier function (5, 10). The skin microbiome may influence epidermal development and cutaneous immune responses (11, 12), and Kumarasinghe et al. proposed that overgrowth of anaerobic flora could contribute to hyperkeratotic flexural eruptions resembling GP (13). GP has also been reported in association with chemotherapy for ovarian cancer (14).

Differential diagnosis: The clinical presentation of GP may be confused with several pustular and inflammatory dermatoses (1). Pustular psoriasis may present with widespread sterile pustules on an erythematous, infiltrated background and, in severe cases, systemic symptoms such as fever. Histopathology typically shows psoriasiform epidermal changes and neutrophilic collections rather than the characteristic granular parakeratosis seen in this case (2). Superficial fungal infections may present with erythema and scaling and can clinically mimic GP. In superficial fungal infections, direct microscopic examination typically demonstrates fungal hyphae, and PAS staining may reveal fungal elements within the stratum corneum. In the present case, however, repeated fungal microscopic examination showed no hyphae or spores, and PAS staining of lesional tissue was negative, providing no evidence of superficial fungal infection (3). IgA pemphigus may present with superficial pustules in annular or circinate configurations; direct immunofluorescence typically demonstrates intercellular IgA deposition in the epidermis, which was absent in this case (4). Subcorneal pustular dermatosis is characterized by recurrent superficial pustules with subcorneal neutrophilic accumulation on histopathology; the presence of characteristic granular parakeratosis supported GP in this patient. Inverse psoriasis, seborrheic dermatitis, and erythrasma were also considered less likely on the basis of the clinical and ancillary findings.

The prognosis of GP is generally favorable. Removal of suspected irritants and measures to restore the skin barrier may lead to remission, whereas selected persistent cases have been treated with topical or systemic retinoids (15–17). In the present case, complete remission occurred after discontinuation of the BAC-containing sanitizer, repeated rinsing of clothing, and topical emollient therapy. Because these interventions were introduced simultaneously and no confirmatory challenge testing was performed, the clinical response supports an association with irritant avoidance but does not establish BAC as the sole causal agent.

In conclusion, this case highlights BAC-containing laundry sanitizer as a potential trigger of generalized GP. Careful assessment of household product exposure may be useful in patients with GP, particularly when lesions involve flexural or clothing-covered areas. Avoidance of suspected irritants and skin-barrier repair should be considered as practical management measures. Further studies are required to clarify the causal relationship between BAC exposure and GP.

## Limitations

This report has several limitations. First, it describes a single retrospective case and therefore provides limited evidence regarding causality or generalizability. Second, neither patch testing nor a re-challenge test was performed; consequently, BAC should be regarded as a potential trigger rather than a confirmed cause. Third, the commercial laundry sanitizer contained ingredients other than BAC, and a contribution from other components cannot be excluded. Fourth, several interventions—discontinuation of the sanitizer, repeated rinsing of clothing, and topical emollient therapy—were initiated concurrently, making it impossible to determine the independent effect of each measure. Further controlled studies incorporating confirmatory testing are needed to clarify the relationship between BAC-containing laundry products and GP.

## Data Availability

The datasets presented in this article are not readily available because the datasets contain clinical information from an individual patient and are not publicly available due to privacy and ethical restrictions. Relevant anonymized data may be made available by the corresponding author upon reasonable request, subject to applicable institutional and privacy requirements. Requests to access the datasets should be directed to Liang Zhang, xiaokela2009@126.com.
